# Efficiency Analysis of Hemostatic Agents in Drainless Neck Surgery: Cellulose-Based Versus Liquid Fibrin Sealants

**DOI:** 10.7759/cureus.62147

**Published:** 2024-06-11

**Authors:** Omar Majadla, Jacob Pitaro, Haim Gavriel, Limor Muallem Kalmovich

**Affiliations:** 1 Otolaryngology - Head and Neck Surgery, Shamir Medical Center (Assaf Harofeh), Be’er Ya’akov, ISR; 2 Faculty of Medical and Health Sciences, Tel Aviv University, Tel Aviv, ISR

**Keywords:** fibrin sealant, length of stay, postoperative, seroma, hemostatic agents, drainless surgery, head and neck surgery

## Abstract

Purpose: Using liquid fibrin sealants has once again questioned the benefit of drain placement in head and neck operations. Cellulose-based hemostats offering different hemostasis mechanisms have scarcely been investigated in drainless neck surgeries. This study aimed to evaluate whether liquid fibrin sealant offers any advantage over cellulose-based hemostats in various head and neck surgeries.

Methods: A prospective trial of patients who underwent various neck surgeries between 2020 and 2022. Baseline characteristics and postoperative outcomes were compared between the drain-placed and the drainless groups, with the latter sub-categorized into three groups: fibrin sealant, cellulose-based hemostats, and a combination of both.

Results: A total of 119 patients were included (63 thyroidectomies, 40 parathyroidectomies, and 16 sialoadenectomies). Fifty eight had a drain placed and 61 had no drain. In the drainless group, 23 patients received cellulose-based absorbable hemostats (SURGICEL®/ FIBRILLAR™); 18 patients had fibrin sealants (EVICEL®/TachoSil®/TISSEEL); in 16, a combination of both was used; and in four patients, no hemostatic agent was used. Three (5%) of the 61 drainless patients developed a seroma compared to one (2%) seroma in the drain-placed patients. No advantage was demonstrated using a combination of FIBRILLAR™ with a fibrin sealant nor for any used separately. Drain placement delayed patient discharge by at least one day compared to the group without a drain (p < 0.001).

Conclusion: Drain placement offered a minor advantage in the postoperative course reducing rates of seroma formation, while delaying patient discharge by at least one day. There was no advantage in using a specific hemostatic agent over the other.

## Introduction

Percutaneous drains are commonly used in head and neck surgeries to prevent hematoma and seroma formation by eliminating dead space. However, their use can lead to various complications, such as infection, fistulae formation, discomfort, pain, psychosocial implications, and fluid accumulation due to blockage.

In the past decade, many researchers have questioned whether a routine drain placement in head and neck operations is necessary. The evolution of remote access thyroidectomies without the application of a drain has further strengthened the drainless approach in open surgery. In their meta-analysis on the role of wound drains after thyroid surgery, Woods et al. showed that drains were not routinely required but also increased the infection rate, postoperative pain, and length of hospital admission [1].

In everyday clinical practice, different local hemostatic agents are used to prevent bleeding. The type of local hemostatic agent applied depends on the surgery type, bleeding risk, surgeon’s experience and preference, and product price and availability.

There are various mechanisms through which local hemostasis can stop bleeding. Some improve primary hemostasis, while others stimulate fibrin formation or inhibit fibrinolysis. Fibrin sealants (FSs) are applied to the raw surfaces of the surgical wound before closure and are considered an important adjunct in achieving hemostasis and healing. FSs mimic the ﬁnal common pathway of the clotting cascade, where thrombin cleaves fibrinogen to form a fibrin clot. They are commonly used to aid in hemostasis by sealing small vessels and closing dead space adhering to the wound surfaces, both essential steps in healing following surgery. The use of FSs has been shown to reduce postoperative secretion and subsequent length of hospital stay (LOS) in different types of head and neck surgeries, such as thyroid and parotid surgeries [2-5]. The oxidized cellulose hemostatic patch adheres to the bleeding site, constricting and initiating platelet activation and aggregation, reinforcing the fibrin clot [6]. SURGICEL® (Ethicon, Johnson & Johnson, USA) is an oxidized cellulose hemostatic agent that controls bleeding during surgical procedures.

This study aims to compare postoperative complications, such as seroma formation and LOS in patients who underwent various draineless head and neck surgeries and in whom different hemostatic agents were used compared to patients with a drain and no hemostatic agent. A secondary objective was to examine whether FSs offer any advantage over cellulose-based hemostatics or their combination.

## Materials and methods

Study description

A prospective trial was performed in the Department of Otolaryngology-Head and Neck Surgery, Shamir Medical Center, a single tertiary university-affiliated center in Israel, between 2020 and 2022. The institutional review board (IRB) approved the trial (number 0127-19-ASF), with clinical trial registration number MOH_2020-05-21_008964.

The inclusion criteria for enrollment were patients over 18 years of age who underwent elective head and neck surgeries at our otolaryngology-head and neck surgery department, including hemithyroidectomy or total thyroidectomy for different pathologies, parathyroidectomy, sialoadenectomy, and consent to participate. Excluded from the study were patients with blood clotting disorders and patients who needed neck dissection and revision surgery, such as completion thyroidectomy. The study population included two groups: the first group included drain-placed patients, and the second group without drain. In addition, the drainless surgery group was sub-categorized into three groups: fibrin sealant versus cellulose-based hemostats versus a combination of both. We compared the two study groups' complication rate, LOS, and next-day discharge. Baseline characteristics and postoperative outcomes were compared between the drain-placed and no-drain groups.

Anti-aggregation and anti-coagulation therapy were held before surgery for at least five days, according to the relevant specialist consultant. Postoperative drain secretions were measured daily, and drains were removed when daily volume was ≤25 ml per 24 hours. All patients had their drains removed before discharge from the hospital.

Patient recruitment

Participants were recruited during their pre-surgical evaluation. They were offered to participate and, if agreed upon, provided written informed consent. This stage involves selecting and enrolling patients who meet the eligibility criteria for the clinical trial. Three fellowship-trained consultant otolaryngology surgeons performed the surgeries. Each surgeon had their operation strategy and preference for using a drain at the end of the surgery. One of the surgeons performed only drainless operations, while the other two used a drain in all surgeries. The decision of which surgeon to assign to a particular operation was made randomly. Only the first author (O.M.) knew every participant involved in the study.

Sample size calculation

The sample size for the study was determined based on the work by Reerds et al. [7], with the aim of achieving a power of 80% and an alpha level of 0.05. To assess a 20% difference between groups with a significance level of 0.05, a two-sample t-test was conducted.

Statistical analyses

For the descriptive analysis, categorical data were described using frequencies and percentages. Continuous variables with normal distribution were presented as means ± standard deviations. Median values and ranges were presented for variables that did not meet the standard distribution assumption.

For inferential analysis, categorical variables were compared using the appropriate Chi-square or Fisher's exact tests. Continuous variables were compared between the groups using the independent t-test or Wilcoxon rank-sum test according to the distribution of variables. For variables with a normal distribution, the independent t-test was presented.

P < 0.05 was considered significant. IBM SPSS Statistics for Windows, Version 27.0 (released 2020, IBM Corp., Armonk, NY) was used for the statistical analysis.

## Results

A total of 119 patients were included. Of them, 58 (49%) were in the drain group and 61 (51%) in the drainless group; in the latest group, four patients (3%) did not receive either a drain or a hemostatic agent. Table 1 presents the patient characteristics. No significant differences were found in patient age (p = 0.58), surgery type (p = 0.625), or gender (p = 0.064).

**Table 1:** Comparison of patients' characteristics and types of surgeries between the drain and drainless groups.

Variable	Drain group (n = 58)	Drainless group (n = 61)	P-value
Mean age, years ± standard deviation	56.1±15.9	54.6 ± 14.3	0.582
% Male (n)	33 (19)	18 (11)	0.064
% Female (n)	67 (39)	82 (50)	0.064
% Parathyroidectomy (n)	35 (20)	33 (20)	0.625
% Thyroidectomy (n)	55 (32)	51 (31)	
% Sialoadenectomy (n)	10 (6)	16 (10)	

The following surgeries were performed: parathyroidectomy, thyroidectomy, parotidectomy, and submandibular gland excision. Of the thyroidectomy surgeries, seven total thyroidectomies and 24 hemithyroidectomies were performed in the drainless group, while no total thyroidectomy was performed in the drain group.

Comparing drain and drainless groups

One patient (2%) in the drain group had seroma formation following submandibular gland excision, while three patients (5%) in the drainless group had seroma formation, one following hemithyroidectomy, one following total thyroidectomy, and one post submandibular gland excision. However, this difference was not statistically significant (p = 0.619). All cases of seroma were treated with a single needle aspiration and prophylactic antibiotic course, and there were no further complications.

Four complications were observed in the drain group (7%) (p = 0.05): one patient had an infection in the surgical bed after thyroidectomy, two patients had swelling in the surgical bed with no detected seroma on examination, and one patient experienced bleeding from superior thyroid artery immediately after the operation and was taken back to operation room from the recovery room. (Table 2).

**Table 2:** Comparison of postoperative complications and course between drain and drainless groups * postoperative bleeding, surgical site infection (SSI)

Variable	Drain	No drain	P-value
% Seroma (n)	2 (1)	5 (3)	0.619
% Other complications (n)*	7 (4)	0 (0)	0.053
Median length of hospital stay, days [range]	2 [2-3]	1 [1-2]	<0.001
% Next day discharge (n)	17 (10)	72 (44)	<0.001

**Figure 1:**
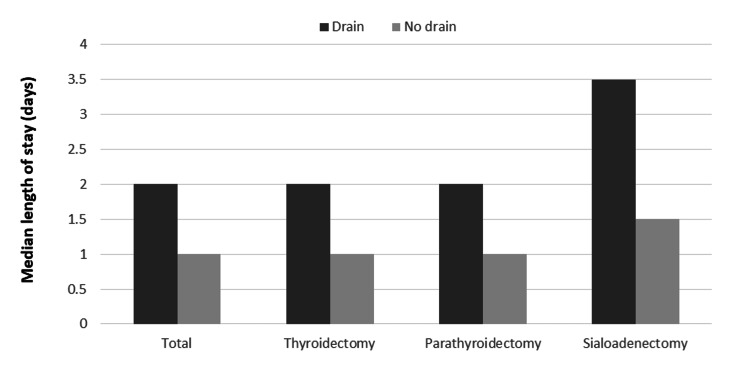
Length of hospital stay by surgery type

According to Figure 1, the median LOS was two days in the drain group and only one day in the drainless group when comparing both groups based on the type of surgery. Even when considering the different types of surgeries, this difference was statistically significant (p < 0.001).

**Figure 2:**
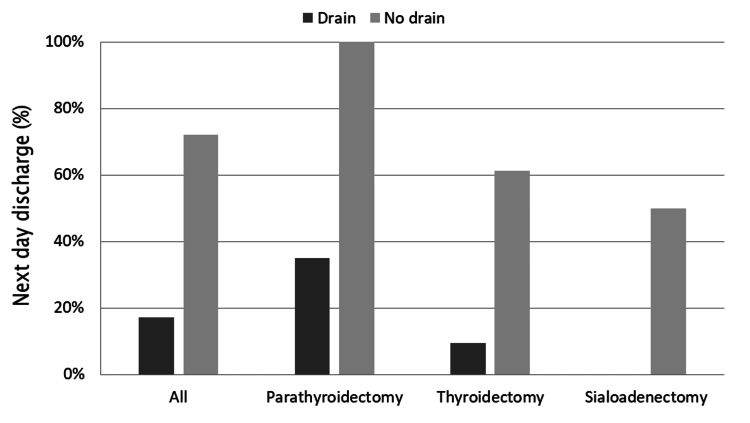
Next-day discharge outcomes by surgery type.

The rate of next-day discharge is illustrated in Figure 2, categorized by the type of surgery.

Patients who underwent parathyroidectomy and thyroidectomy without drain had significantly higher next-day discharge rates than those with drain (100% vs. 35%, p < 0.001 and 61% vs. 9%, p < 0.001, respectively). A significantly higher rate of discharge on POD1 was noted in the drainless group regardless of the type of surgery compared to the drain group (72% vs. 17%, p < 0.001).

Comparing dry versus liquid hemostatic agents

The efficacy and postoperative course of various hemostatic agents in the drainless group are presented in Table 3.

**Table 3:** Comparison between different types of hemostatic agents used

Variable	Cellulose-based (n = 23)	Fibrin sealant (n = 18)	Both (n = 16)	P-value
Mean age, years, ± standard deviation	57.1 ± 13.4	55.1 ± 14.2	49.6 ± 16.2	0.143
% Male (n)	22 (5)	17 (3)	13 (2)	
% Female (n)	78 (18)	83 (15)	88 (14)	
% Parathyroidectomy (n)	44 (10)	28 (5)	25 (4)	<0.001
% Sialoadenectomy (n)	0 (0)	39 (7)	0 (0)	
% Thyroidectomy (n)	57 (13)	33 (6)	75 (12)	
% Complications (n)	0 (0)	0 (0)	0 (0)	
% Seroma (n)	0 (0)	6 (1)	13 (2)	0.185
Median length of hospital stay, days [range]	1 [1-2]	1 [1-2]	1 [1-1]	0.174
% Next day discharge (n)	74 (17)	56 (10)	81 (13)	0.256

The drainless group was divided into three subgroups by the type of agent used: 23 patients had cellulose-based absorbable hemostats (SURGICEL®/FIBRILLAR™) (40%), 18 had fibrin sealants (EVICEL® (Ethicon, Johnson & Johnson Surgical Technologies, USA)/TachoSil® (Corza Medical, USA)/TISSEEL (Baxter International Inc., USA)) (31%) and 16 with a combination of both (28%). Seroma was observed in three patients (5%): one in the fibrin sealant group post submandibular gland excision and two patients in the combination group following total thyroidectomy and hemithyroidectomy. LOS and next-day discharge rates were similar across all groups (p < 0.17 and p < 0.26, respectively).

## Discussion

Study outcomes

The use of drains in head and neck surgeries shows no advantages over not using drains. Using a drain while delaying patient discharge by at least one day does not significantly reduce the rate of seroma formation. No significant difference was found when using cellulose-based hemostatic (FIBRILLAR™/SURGICEL®) versus fibrin sealants (EVICEL®/TachoSil®/TISSEEL) or using both agents together.

Interpretation

Placing a drain in head and neck surgery is a common practice, but our focus expands toward the quality of life rather than safety alone, drainless surgery is increasingly reported in head and neck surgery, mainly in thyroid surgery [1,2,6,8,9], with a simultaneous increase in the use of fibrin sealants, as well as in parotidectomy and neck dissection surgeries [4,5,7,10-14].

Our study adds weight to this evolving practice by demonstrating favorable outcomes associated with drainless surgeries, as our study findings showed no significant postoperative complications, shorter hospital stays, increased patient satisfaction, and cost savings for healthcare systems.

Our findings revealed that there was no significant advantage in using dry, liquid, or a combination of both hemostatic agents. In comparison to Al-Qahtani's [13] study, which had a small sample size focusing solely on parotidectomy and reported two complications including temporary facial nerve palsies, our study encompassed a broader range of surgeries, both benign and malignant diseases, and offered a more comprehensive perspective with a larger study sample size.

Therefore, our study results are highly relevant in assessing the efficacy of various hemostatic agents and can be used to guide clinical decisions in surgical settings.

Our prospective study offers a wider perspective and involves different surgeries and indications for surgery, including both benign and malignant diseases, in contrast to Cohen et al. [14], who recently conducted a retrospective study on the impact of drainless head and neck surgeries and the use of fibrin sealant in neck dissections in focusing on malignant diseases only.

Spotnitz [15] reviewed fibrin sealant, and one of the themes was preventing seroma formation using fibrin sealant in surgical procedures concluding conflicting evidence on the effectiveness of fibrin sealant as an improper application can result in non-adherent fibrin sealant, which may worsen seroma formation. Hence, fibrin sealant for seroma prevention is still debated. We conducted a prospective clinical trial to evaluate the effectiveness of fibrin sealant in various head and neck surgeries. When comparing fibrin sealant to cellulose-based agents, we found no significant postoperative seroma formation among those who received fibrin sealant.

Strengths and limitations

This study has several strengths. First, it is a prospective study with a large group of participants. Second, it includes a variety of different types of surgeries under the two categories mentioned above. This wider range of surgeries provides a more diverse set of outcomes. Furthermore, all surgeries were performed by consultant ENT surgeons specializing in head and neck surgery.

We acknowledge the limitations of our study. First, we did not actively measure seromas in all patients after the surgery. Instead, we only drained patients who reported dissatisfaction or visited the emergency room with swelling in the surgical area. This means that there could have been more seromas than reported, but they did not have any clinical implications. Second, our study did not compare each patient's histology or consider the thyroid's size removed. However, we plan to conduct further research in this area in the future. Third, a possible bias could be caused by the surgeon's preferences, as each surgeon brings their unique approach, experience, and preferences, which are a natural part of the process. Comparing two groups of patients for each surgeon was a possibility, but our concern was that subdividing the cases among surgeons for analysis would result in groups that were too small to achieve statistical power.

## Conclusions

Placing a surgical drain in head and neck surgeries may prevent seroma formation. However, it may also delay patient discharge by up to two days. There was no significant difference in the effectiveness of cellulose-based hemostatic agents like SURGICEL®/FIBRILLAR™ and fibrin sealants such as EVICEL®/TachoSil®/TISSEEL. The surgeon should decide to use a drain or hemostatic agent based on their judgment and intraoperative considerations.
